# Super Enhancer Regulatory Gene FYB1 Promotes the Progression of T Cell Acute Lymphoblastic Leukemia by Activating IGLL1

**DOI:** 10.1155/2023/3804605

**Published:** 2023-09-19

**Authors:** Kunlong Zhang, Jun Lu, Fang Fang, Yongping Zhang, Juanjuan Yu, Yanfang Tao, Wenyuan liu, Lihui Lu, Zimu Zhang, Xinran Chu, Jianwei Wang, Xiaolu Li, Yuanyuan Tian, Zhiheng Li, Qian Li, Xu Sang, Li Ma, Ningling Wang, Jian Pan, Shaoyan Hu

**Affiliations:** ^1^Children's Hospital of Soochow University, Suzhou 215003, China; ^2^Department of Pediatrics, The Second Affiliated Hospital of Anhui Medical University, Hefei 230601, China; ^3^Department of Hematology, Children's Hospital of Soochow University, Suzhou 215003, China; ^4^Institute of Pediatric Research, Children's Hospital of Soochow University, Suzhou 215003, China

## Abstract

**Background:**

Arising from T progenitor cells, T-cell acute lymphoblastic leukemia (T-ALL) is an aggressive hematologic malignant tumor, accounting for 15% of childhood ALL and 25% of adult ALL. Composing of putative enhancers in close genomic proximity, super enhancer (SE) is critical for cell identity and the pathogenesis of multiple cancers. Belonging to the cytosolute linker protein group, FYB1 is essential for TCR signaling and extensively studied in terms of tumor pathogenesis and metastasis. Dissecting the role of FYN binding protein 1 (FYB1) in T-ALL holds the potential to improve the treatment outcome and prognosis of T-ALL.

**Methods:**

In this study, SEs were explored using public H3K27ac ChIP-seq data derived from T-ALL cell lines, AML cell lines and hematopoietic stem and progenitor cells (HSPCs). Downstream target of FYB1 gene was identified by RNA-seq. Effects of shRNA-mediated downregulation of FYB1 and immunoglobulin lambda-like polypeptide 1 (IGLL1) on self-renewal of T-ALL cells were evaluated *in vitro* and/or *in vivo*.

**Results:**

As an SE-driven gene, overexpression of FYB1 was observed in T-ALL, according to the Cancer Cell Line Encyclopedia database. *In vitro*, knocking down FYB1 led to comprised growth and enhanced apoptosis of T-ALL cells. *In vivo*, downregulation of FYB1 significantly decreased the disease burden by suppressing tumor growth and improved survival rate. Knocking down FYB1 resulted in significantly decreased expression of IGLL1 that was also an SE-driven gene in T-ALL. As a downstream target of FYB1, IGLL1 exerted similar role as FYB1 in inhibiting growth of T-ALL cells.

**Conclusion:**

Our results suggested that FYB1 gene played important role in regulating self-renewal of T-ALL cells by activating IGLL1, representing a promising therapeutic target for T-ALL patients.

## 1. Introduction

As an aggressive hematological tumor, T-cell acute lymphoblastic leukemia (T-ALL) accounts for 15% and 25% of childhood and adult ALL, respectively, and presented poor sensitivity to chemotherapy drugs and prone to the drug resistance [1–4]. About 15% of childhood T-ALL and 40% of adult T-ALL will relapse eventually with subsequent poor prognosis after extensive combination chemotherapy [5, 6]. Therefore, it is conducive to explore the pathogenesis of T-ALL to achieve better treatment and prognosis.

As a large cluster composed of several adjacent near-ordinary enhancers, super enhancer (SE) is commonly observed in high-density transcription factors, cofactors, and enhancer-related epigenetic modifications. By regulating lineage-specific genes and oncogens, SE is critical for cell identity and the pathogenesis of multiple cancers [7–10]. Inhibiting the activity of SE may significantly comprised the growth and survival of tumor cells, and exploring roles of SE-driven genes may hold great potential for developing targeted therapies [11].

The FYN binding protein 1 (FYB1) encodes the adhesion and degranulation-promoting adapter protein (ADAP) and is highly expressed in T cells, NK cells, bone marrow cells, and platelets, responsible for the signal transduction upon T cell receptor (TCR) stimulation with integrin activation [12].

Studies have shown key roles of FYB1 gene in megakaryocyte-specific platelet production [13] and autoimmune encephalomyelitis [14]. In addition, deficiency of FYB1 can enhance the cytotoxicity of CD8+ CTL and inhibit the expression of PD-1 in CD8+ effector T cells, thus significantly inhibiting tumor growth and enhancing antitumor immunity [15]. High expression of FYB1 was reported in various types of breast cancers, accounting for the recurrence and metastasis of this disease [16]. Of note, aberrant high expression of FYB1 was suggested as a biomarker for poor prognosis in advanced cutaneous T-cell lymphoma [17]. However, the role of FYB1 gene in T-ALL remains poorly understood.

In this study, we identified FYB1 as an SE-driven gene using public H3K27ac ChIP-seq data from T-ALL cell lines, acute myeloid leukemia (AML) cell lines and hematopoietic stem and progenitor cells (HSPCs). As a SE-driven gene, FYB1 gene was highly expressed in T-ALL cell lines, suggesting plausible dependance of T-ALL cells on the high expression of FYB1 gene. Downregulating FYB1 blunted the growth of T-ALL cells both *in vitro* and *in vivo* through the downstream effector, IGLL1. Our results proposed FYB1 as a potential vulnerability of T-ALL cells, representing an interesting therapeutic target.

## 2. Materials and Methods

### 2.1. Cell Line and Culture

Human T-ALL cell lines, including HUT78, J-gamma1, CCRF-CEM, MOLT-4, Jurkat and 6T-CEM, human AML cell lines, including MV4-11 and NB4, and human CML cell line K562, were all cultured with Roswell Park Memorial Institute (RPMI)-1640 medium (Biological Industries; Sartorius AG) supplemented with 10% FBS (Biological Industries, CT, USA) and 1% penicillin–streptomycin (Millipore Sigma, MA, USA). Human neuroblastoma cell lines SK-N-BE2 and IMR32, human gastric cancer cell lines OACP4C and HGC-27, and human osteosarcoma cell line HOS were all cultured with DMEM medium (Biological Industries; Sartorius AG) supplemented with 10% FBS (Biological Industries, CT, USA) and 1% penicillin–streptomycin (Millipore Sigma, MA, USA). All cell lines mentioned above were purchased from the Chinese Academy of Sciences Cell Bank and were cultured in a humidified incubator with 5% CO_2_ gas at 37°C and subjected to routine mycoplasma testing. In 2020 and 2021, all cell lines were confirmed by short tandem replicates.

### 2.2. Cell Proliferation and Viability Measurement

Cells (J-gamma1, 6T-CEM and Jurkat) were plated in a 96-well plate with a density of 1 × 10^3^. Following the instructions of the manufacturer, Cell Counting Kit-8 (CCK8) (Dojindo Molecular Technologies, Tokyo, Japan) was applied to determine the cell viability. Community multiplication was computed as a percentage of cell growth in the control medium. Cell density was measured three times and repeated in at least three separate trials. Plots and statistic analysis were performed by Graph Prism software 8.4.3 (GraphPad Software, Inc., San Diego, CA, USA).

### 2.3. RNA Preparation and Real-Time PCR Expression Analysis

The quantitative real-time polymerase chain reaction (qRT-PCR) was conducted as mentioned previously [18]. TRIzol® reagent (Invitrogen, CA, USA) was used to extract total RNA. The high volume cDNA Reverse Transcription Kit (Applied Biosystems, CA, USA) was used to synthesize cDNA from 2 mg total mRNA. qRT-PCR was performed on LightCycler 480 real-time system (cat. No. 04707516001; Roche, Penzberg, Germany). GAPDH was used as an internal control, the mRNA expression level was calculated by the *Δ*CT method. Sequences of primers used in this study were shown as below: GAPDH: forward: TGCACCACCAACTGCTTAG, reverse: GATGCAGGGATGATGTTC, FYB1 forward: GGATGTCTCAGTCAATAGCCG, and reverse: GGTTCCTTGTCAGGCTTTTCC.

### 2.4. Preparation and Infection of Lentivirus

shRNAs targeting FYB1 and IGLL1 (sequences were shown in Table 1) were cloned into the pLKO.1-puro lentivirus vector (IGE BIOTECHNOLOGY LTD, Guangzhou, China). HA-tagged FYB1 CDS sequence was cloned into the pLKO.1-puro lentivirus vector. (IGE BIOTECHNOLOGY LTD, Guangzhou, China). High titer of lentivirus was prepared using pMD2.G and psPAX2 (pMD2. G: #12, 259; psPAX2: #12,260; Cambridge, MA, USA). According to the manufacturer's instructions, polyethyleneimine (linear MW 25,000 Da, 5 mg/ml, pH7.0) (Item No.: 23966-1; Polysciences, Warrington, PA, USA) was used to cotransfect pMD2.G, psPAX2 in 293FT cells. Lentivirus supernatant was collected and filtered using a 0.45 *μ*m filter. Leukemia cells were then incubated with lentivirus for 24 hr supplemented with polyurethane (Sigma–Aldrich) of 10 *μ*g/ml. Puromycin (10 *μ*g/ml) selection was used to make stable T-ALL cell lines was prepared with 10 *μ*g/ml puromycin (Invitrogen; Thermo Fisher Scientific, Inc.).

### 2.5. RNA-Seq and Data Processing

In accordance with the protocol advised by Novogene (Beijing, China), RNA sequencing was carried out as brief described as below. Total RNA was first retro-transcribed into cDNA for library construction, followed by next-generation sequencing. The original reads were then filtered and mapped against HISAT for clean reads. Gene expression levels were then computed (in fragments per kilobase exon model mapped per million reads). The differentially expressed genes (DEGs) were identified by DESeq2 analysis (*P* < 0.05 and Log2 (fold change) >0.5 or Log2 (fold change) <−0.5). The RNA sequencing data have been uploaded to the GEO database (https://www.ncbi.nlm.nih.gov/geo) under the session of GSE197450.

### 2.6. Apoptosis Analysis

Apoptosis was analyzed as mentioned previously [18]. T-ALL cells (6T-CEM and J-gamma1 cell lines) were infected with lentivirus for 24 hr in the presence of 10 *μ*g/ml polyurethane (Sigma–Aldrich). Stable cell lines were selected with 10 *μ*g/ml of puromycin (Invitrogen; Thermo Fisher Scientific, Inc.). Leukemia cells obtained after 5 days of screening were washed with precooled 1x PBS and suspended in a 1x binding buffer and then treated using the FITC-Annexin V Apoptosis Kit (item no.: 556420; BD Biosciences, Franklin Lakes, NJ, USA) according to the manufacturer's instructions. Flow cytometry (Beckman Gallios™ Flow Cytometry; Beckman) was used to analyze the apoptosis.

### 2.7. Cell Cycle Analysis

The cell cycle was analyzed as mentioned previously [18]. The cells were fixed using 70% ethanol at 4°C overnight. After 24 hr, the fixed cells were permeated with 0.5% Triton X-100, stained at 37°C using PI (1.5 *μ*mol/l; Catalog no.: P4170; Sigma–Aldrich; Merck KGaA) and 25 *μ*g/ml RNase A, and kept in dark for 1 hr. Beckman Gallios™ flow cytometry (Immunotech; Beckman Colter, Inc.) was used to evaluate cell cycle distribution. The MultiCycle AV DNA analysis software (Version: 328; Verity Software House, Inc.) was used to analyze the proportion of cells at different stages of the cell cycle.

### 2.8. Western Blotting Analysis

Western blotting was analyzed as mentioned previously [18]. The RIPA lysis buffer (Beyotime Institute of Biotechnology) supplemented with protease and phosphatase inhibitors was used to lyse cells (Jurkat, 6T-CEM and J-gamma1). After ultrasonic treatment, the supernatant was collected using centrifugalization as total protein, and its concentration was quantitatively determined by the BCA kit (Thermo Fisher Science). Western blotting analysis was conducted with reference proteins of the following primary antibodies including FYB1 (cat. ab76103; Abcam), IGLL1 (cat. ab154517, Abcam), PARP (cat. No. 9542; Cell Signaling Technology), cleaved caspase-3 (cat. 9661S, Cell Signaling Technology), caspase-8 (cat. 9746, Cell Signaling Technology), HA (cat. ab9110; Abcam), glyceraldehyde 3-phosphate dehydrogenase (GAPDH) (cat. MA3374; Millipore); Z-VAD-FMK (cat. HY-16658B; MedChemExpress), and incubated using the affiniure IgG (H + L)/biotin and peroxidase (cat. 111-035-003;), and IgG (H + L) (cat. 115-035-003;). All secondary antibodies were purchased from Jackson ImmunoResearch Laboratories, Inc. (West Grove, PA, USA). LAS 4010 (Cytiva) imaging system and ImageQuant TL 8.1 software (Cytiva) were used to observe the protein bands with ECL ultrasensitive luminescent solution (Thermo Fisher Scientific, Inc.); and the ImageJ software was used for band quantification. Then, the GAPDH antibody was used as an internal control.

### 2.9. In Vivo Xenograft Leukemia Model

All animal procedures in this study have been approved and licensed by the Animal Care and Use Committee of Children's Hospital of Soochow University (CAM-SU-AP #: JP-2018-1). Female NSG mice aged 4–8 weeks (NOD-Prkdc) Scid II2rgEm1/Smoc, NM-NSG-001) were purchased from Shanghai Model Organizations. Mice were maintained in a standard SPF room and used in all studies of the sh-NC Group and the sh-FYB1 Group with 12 for each. On Day 0, each mouse was injected with 2 × 10^6^ Jurkat cells via tail vein injection. On the 18th day after cell injection, luciferase was injected into the abdominal cavity of each mouse and followed by immediate anesthesia with isoflurane gas. Then, the NightOWL *in vivo* imaging system (BERTHOLD, Germany) was used for the mouse of each group on the 25th, 30th, and 35th days. Mice weight and fur were monitored every 5 days. Mice were euthanized when they reached the humane endpoint. The liver, spleen, peripheral blood (PB), and bone marrow of mice in the sh-NC group and the sh-FYB1 group were collected; and the liver and spleen organs were subjected for imaging. The flow cytometry (Beckman Gallios™ Flow Cytometry; Beckman) was used to analyze the expression quantity of antihuman CD45+ after grinding the liver, spleen, PB, and bone marrow of mice and the differences between the sh-NC Group and the sh-FYB1 group were compared. Immunohistochemistry and HE (hematoxylin and eosin) staining were conducted for each organ specimen. Cleaved-Caspase 3 (item no.: GB11009-1, 1:30, Servicebio, Boston, MA, USA) and Ki67 (item no.: AB 15580, 1:30, Abcam, Cambridge, UK) were used according to the manufacturer's instructions.

### 2.10. Public ChIP-Seq Data Collection and Analysis

Public ChIP-Seq H3K27ac datasets of T-ALL cell lines, AML cell lines, and HSPCs were downloaded from the Cistrome database (http://www.cistrome.org/). The obtained ChIP-Seq H3K27ac datasets (GSE70734, GSE29611, GSE50622, GSE76783, GSE59657, GSE80779, GSE123872, GSE188750, GSE71809, GSE70660, and GSE93372) were aligned to the reference genome (UCSC hg38) with alignment parameters -p 4 -q -x [19]. Peaks were detected with MACS2 (v2.0.9) [20] for the parameters -g hs -n test -B -q 0.01. The bedgraph files produced by MACS2 were converted to the bigwig files with the UCSC bedGraphToBigWig tool, and then the bigwig files were visualized with the Integrative Genomics Viewer (IGV) [21]. Then we identified SEs by the ROSE (rank order of SEs) method [22, 23], according to the parameters -s 12500 -t 2000 (-s stitching distance; -t TSS exclusion zone size).

### 2.11. Public Database

The expression level of FYB1 and IGLL1 mRNA in different types of tumor cell lines was obtained from the Cancer Cell Line Encyclopedia (CCLE) (http://www.broadinstitute.org/ccle).

### 2.12. Data Statistics and Analysis

All experiments were performed in triplicates and independently repeated at least three times. Statistical analyses were performed with GraphPad Prism 8.4.3 (GraphPad Software, Inc.). *t*-Test or Mann–Whitney *U*-test was used for comparison between the two groups. The *P* values with statistical significance are indicated as  ^*∗*^*P* < 0.05,  ^*∗∗*^*P* < 0.01,  ^*∗∗∗*^*P* < 0.001, and  ^*∗∗∗∗*^*P* < 0.0001.

## 3. Results

### 3.1. Super Enhancers Are Enriched in T-ALL-Associated Oncogenes in T-ALL Cell Lines

**Figure 1:**
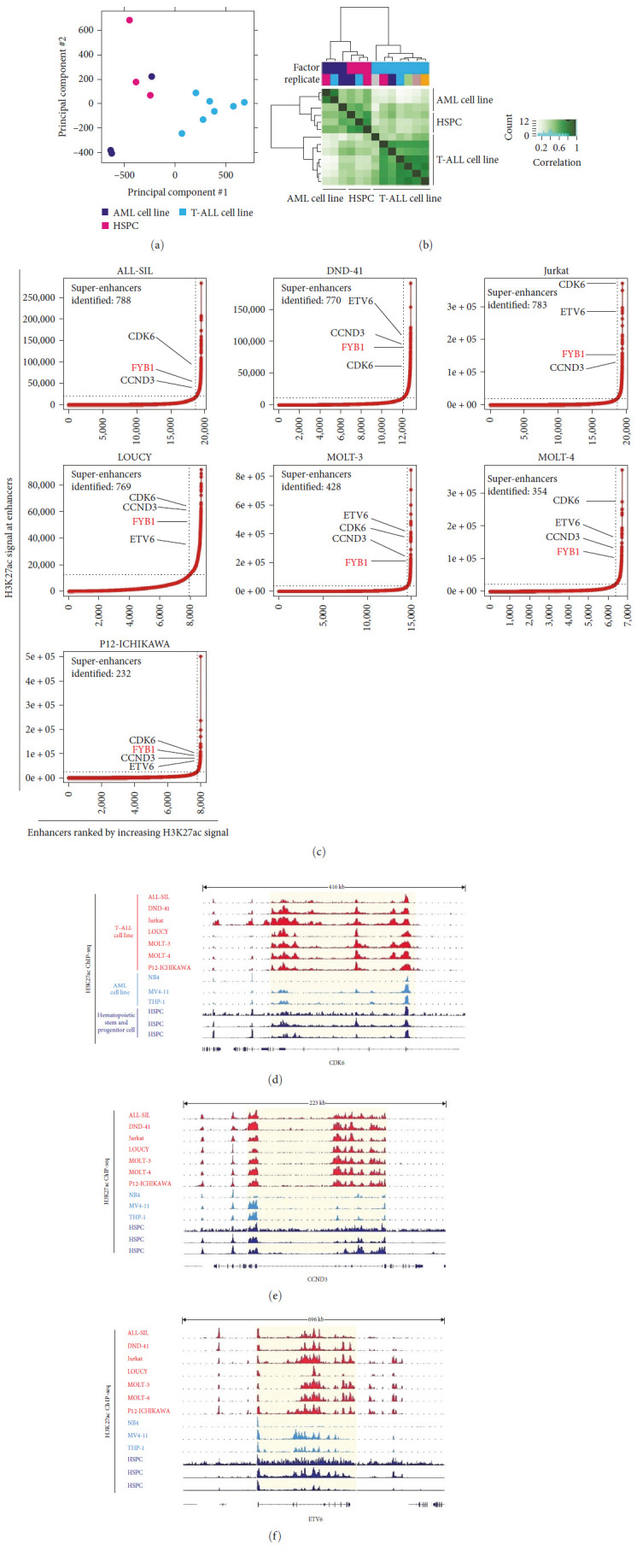
ChIP-Seq H3K27ac datasets analysis of T-ALL cell lines, AML cell lines, and hematopoietic stem and progenitor cells (HSPCs). (a) PCA was performed for 7T-ALL cell lines, three AML cell lines, and three HSPCs based on the H3K27ac signals identified in each sample. Each circle represents a sample, and each color represents the type of sample. (b) Cluster analysis results of 7T-ALL cell lines, three AML cell lines, and three HSPCs based on the H3K27ac signals identified in each sample. (c) Super enhancer profiling in 7T-ALL cell lines. Enhancers were ranked by increasing H3K27ac signal. Number of super enhancers identified in each T-ALL cell line was shown. Examples of genes commonly associated with super enhancers in at least 6T-ALL cell lines were shown. the *x*-axis stands for Enhancers ranked by increasing H3K27ac signal, and the *y*-axis stands for H3K27ac signal at enhancers. (d–f) SEs were identified at the locus of CDK6 (d), CCND3 (e), and ETV6 (f). SE was highlighted by the shaded box. Peaks stand for H3K27ac signals.

To identify the genes correlated with SEs in T-ALL, we analyzed public H3K27ac ChIP-seq datasets in 7T-ALL cell lines (ALL-SIL, DND-41, Jurkat, LOUCY, MOLT-3, MOLT-4, P12-ICHIKAWA). In this study NB4, MV4-11, and THP-1 were also used as representative AML cell lines. Additionally, we included three HSPC) samples, to compare the H3K27ac signals among those T-ALL, AML, and HSPC cell samples. The principal component analysis (PCA) result and clustering result based on the peak signals clearly distinguished T-ALL samples from AML or HSPC (Figures 1(a) and 1(b)). Putative SEs identified in each of the 7T-ALL cell lines are shown in Figure 1(c) and *Supplementary 1*. A total of 213 genes were selected which were commonly correlated with SEs in at least 6T-ALL cell lines, including CDK6, CCND3, ETV6, and FYB1 (Figure 1(c)). As shown in Figure 1(d)–1(f), the enhancer region of T-ALL-associated oncogene CDK6, CCND3, and ETV6 in T-ALL cell lines showed coincident H3K27ac signals that were not present in AML or HSPC cells. Our results indicate that SE was commonly seen in T-ALL associated genes.

### 3.2. FYB1 Was Activated by Super Enhancer and Highly Expressed in T-ALL Cell Lines

**Figure 2:**
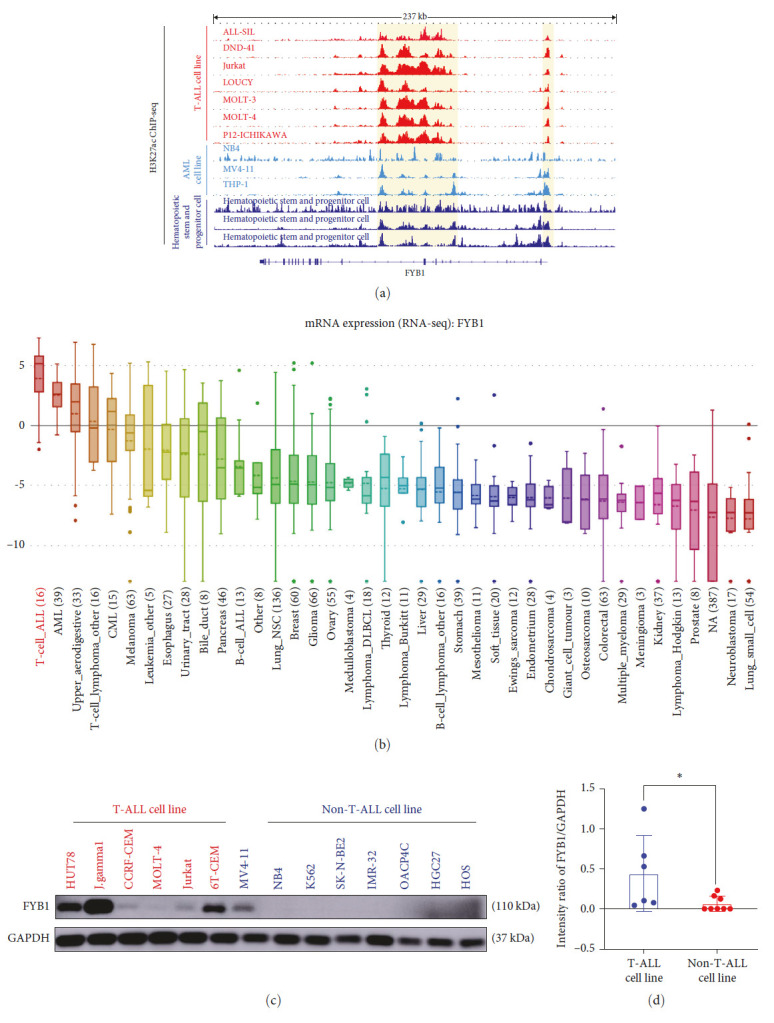
FYB1 was super-enhancer associated and highly expressed in T-ALL cell lines. (a) The ChIP-Seq gene tracks represented the H3K27ac signal in 7T-ALL cell lines, three AML cell lines and three HSPCs at the FYB1 gene locus. The super enhancers were labeled by yellow boxes. (b) According to the CCLE (https://portals.broadinstitute.org/ccle), FYB1 was highly expressed in T-ALL. (c and d) The western blotting results showed that the protein level of FYB1 in T-ALL cells was significantly higher than that in non-T-ALL cells.

FYB1 was identified as one of the SE-driven genes in T-ALL cells (Figure 1(c)). SE was consistently observed at FYB1 locus in all 7T-ALL cell lines but not in either AML cell lines or HSPC cells (Figure 2(a)). In line with this, expressions of FYB1 were highest in T-ALL cell lines among other cancer cell lines, according to the CCLE dataset (https://portals.broadinstitute.org/ccle) (Figure 2(b)). Furthermore, high expression of FYB1 was confirmed by western blot in T-ALL cell lines, HUT78, J-gamma1, CCRF-CEM, MOLT-4, Jurkat, and 6T-CEM but not in non-T-ALL cell lines (NV4-11, NB4, K562, SK-N-BE2, IMR-32, OACP4C, HGC27, and HOS) (Figures 2(c) and 2(d)). Collectively, these results indicated that FYB1 was highly expressed in T-ALL cells potentially driven by SE.

### 3.3. FYB1 Knockdown Inhibits the Proliferation and Promotes the Apoptosis of T-ALL Cells

**Figure 3:**
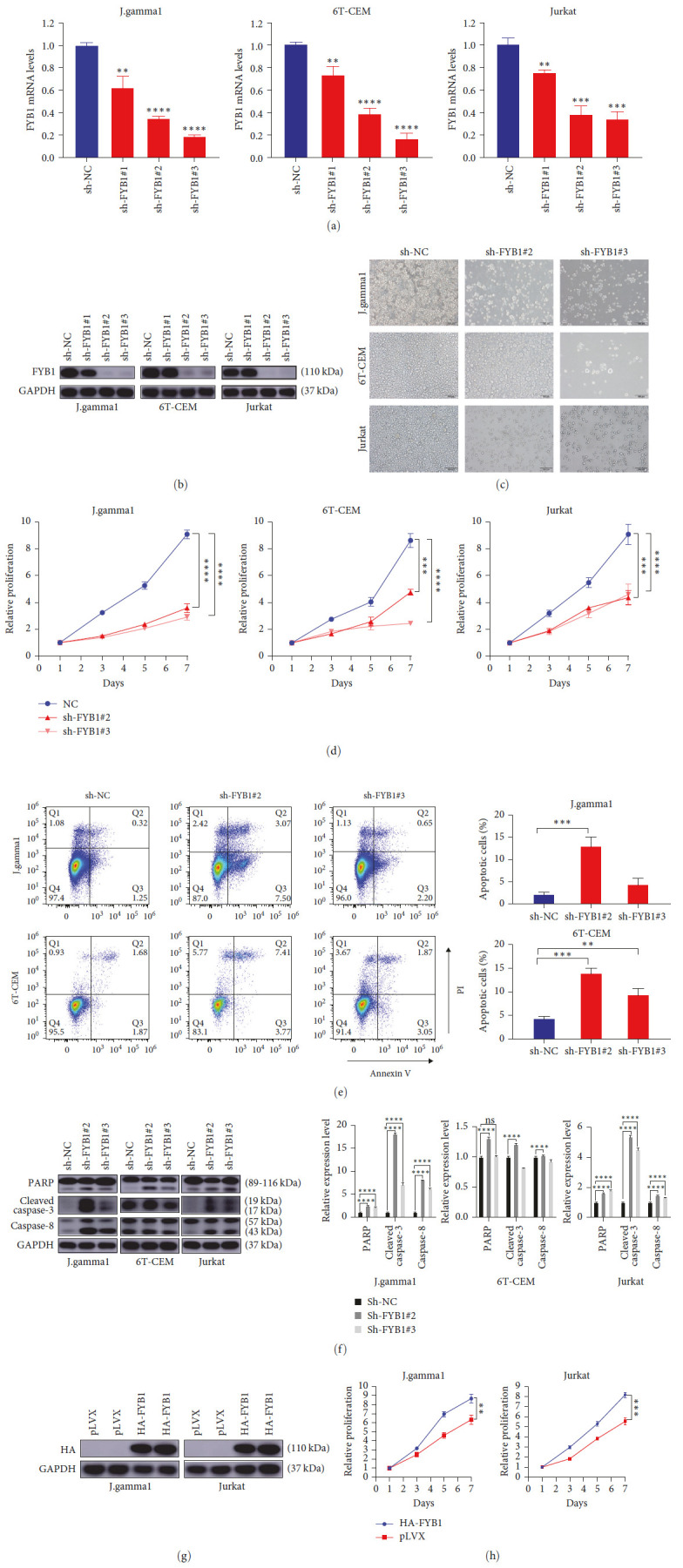
FYB1 knockdown inhibited the proliferation of T-ALL cells *in vitro*. (a and b) shRNA-mediated knockdown efficiency of FYB1 in the J-gamma1, 6 T-CEM and Jurkat cell lines was evaluated by qRT-PCR (a) and western blotting, respectively (b). (c) Downregulation of FYB1 significantly inhibited the proliferation of J-gamma1, 6T-CEM and Jurkat cell lines evaluated by imaging assay. (d) Downregulation of FYB1 significantly inhibited the proliferation of J-gamma1, 6T-CEM and Jurkat cell lines evaluated by cell proliferation assay. (e) Downregulation of FYB1 increased the apoptosis of J-gamma1 and 6T-CEM cells. (f) The cleavage of PARP, cleaved-caspase-3 and caspase-8 were enhanced due to downregulation of FYB1 in the J-gamma1, 6T-CEM and Jurkat cells. (g and h) Overexpression of FYB1 enhanced the proliferation of J-gamma1 and Jurkat cells.

To explore the biological role of FYB1, we knocked down FYB1 genes in three T-ALL cell lines (J-gamma1, 6T-CEM and Jurkat) with high expression of FYB1 by shRNAs (Table 1). Knocking down efficiency was confirmed at both mRNA and protein levels (Figures 3(a) and 3(b)). Downregulation of FYB1 significantly inhibited the proliferation of T-ALL cells (*P* < 0.001) (Figures 3(c) and 3(d)). In addition, downregulation of FYB1 led to enhanced apoptosis of J-gamma1 and 6T-CEM cell lines (Figure 3(e)). In consistent with this, elevated levels of PARP and cleaved caspase-3 and -8 were observed as a result of downregulating FYB1 (Figure 3(f)), which can be blocked by apoptosis inhibitor Z-VAD (*Supplementary 2*). In contrast, overexpression of FYB1 resulted in enhanced proliferation of J-gamma1 and Jurkat cells (Figures 3(g) and 3(h)). Taken together, our results suggested that FYB1 was essential for the survival of T-ALL cells.

### 3.4. FYB1 Knockdown Inhibits the Progression of Leukemia in the Xenotransplantation Model

**Figure 4:**
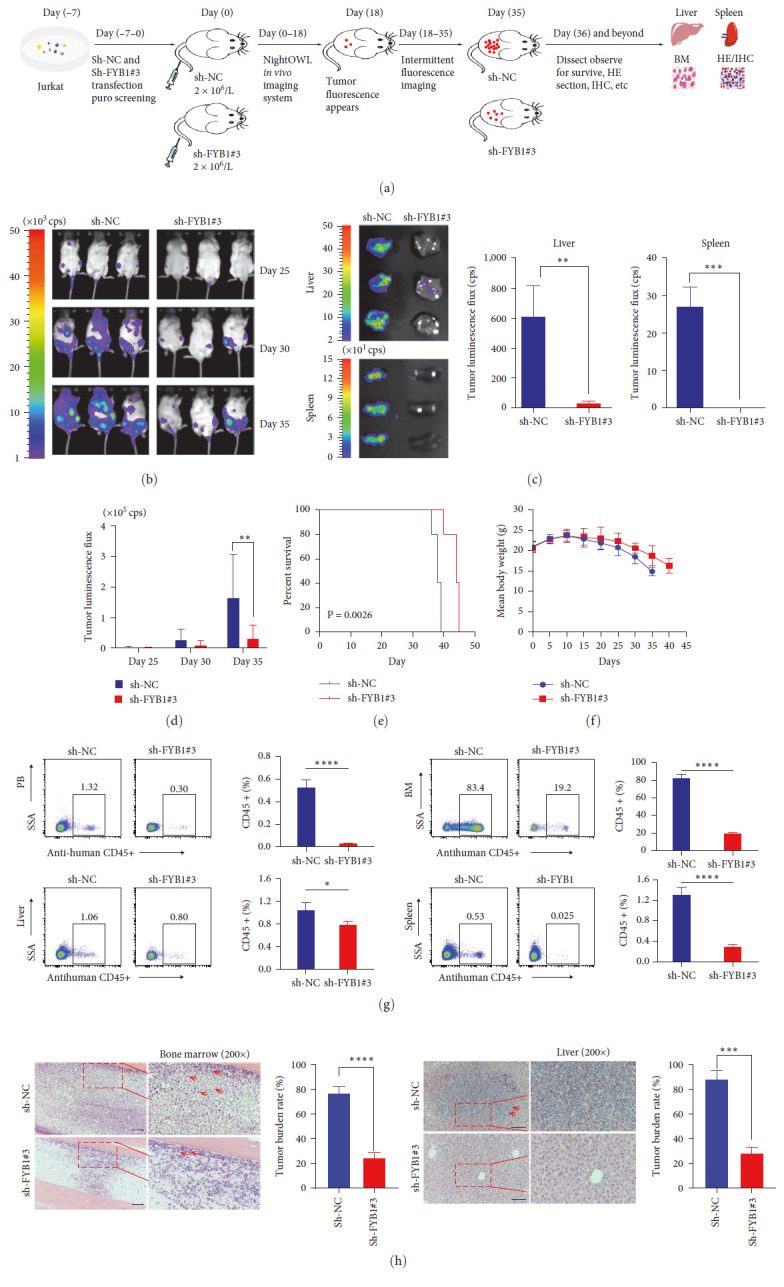
FYB1 knockdown inhibited the growth of T-ALL cells *in vivo*. (a) Schematic diagram of *in vivo* experiments. (b, d) Jurkat cells expressing luciferase transfected with control or FYB1 knockdown were injected into Nod–Scid mice via tail vein injection; and the Night OWL *in vivo* imaging system was used to monitor the leukemia burden of each group on the 25th, 30th, and 35th day after injection. Quantified results of fluorescence were shown by the bar graph in panel D. (c) Fluorescence of livers and spleens from control and FYB1 knockdown groups was determined, and quantified results were shown by the bar graph. (e) Downregulation of FYB1 resulted in better survival rate compared to control group. *P* = 0.0026. (f) No significant difference of body weight was observed between control and FYB1 knockdown group. (g) Percentages of Jurkat cells in liver, peripheral blood (PB), bone marrow (BM), and spleen from control and FYB1 knockdown groups were determined by hCD45 flow cyteometry and quantified results were shown by the bar graph. (h) Representative HE staining of bone marrow and liver in control and FYB1 knockdown groups (zoom factor: 200). Scale bars: 100 *μ*m.

Effects of inhibiting FYB1 expression have been evaluated *in vitro*, we therefore proceeded to evaluate this *in vivo*. To this end, Jurkat cells with or without knocked-down FYB1 were transplanted into Nod–Scid mice (Figure 4(a)). Leukemia burden was determined by *in vivo* fluorescence imaging assay. As shown in Figures 4(b) and 4(d), knocking down FYB1 significantly decreased the expansion of Jurkat cells *in vivo* at three time points, compared to control. In line with this, the fluorescence in the liver and spleen of mice transplanted with FYB1 KD Jurkat cells was significantly lower than control (Figure 4(c)). And percentage of Jurkat cells with knocked-down FYB1 was significantly lower in PB, liver, spleen, and bone marrow when compared to control (Figure 4(g)). Besides, HE staining also showed much less of Jurkat cells with knocked-down FYB1 in the bone marrow and liver (Figure 4(h)). Moreover, knocking down FYB1 led to better survival rate (43 ± 2.0 days) than control (38 ± 1.2 days) (*P* = 0.0026) (Figure 4(e)). No obvious loss of body weight was observed (Figure 4(f)). All the above results suggested that knocking down FYB1 led to a marked delay in leukemia progression *in vivo*.

### 3.5. FYB1 Activates IGLL1 in T-ALL Cell Lines

**Figure 5:**
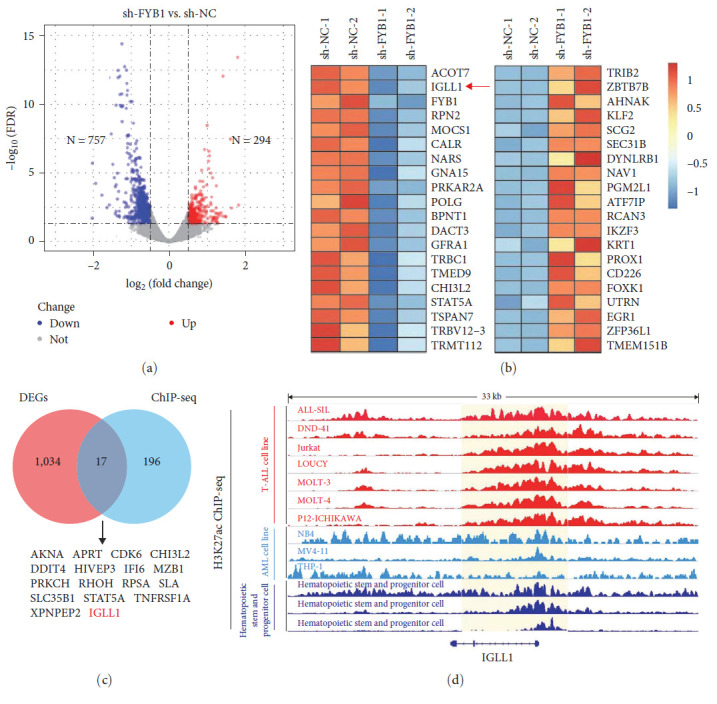
FYB1 activated IGLL1 in the T-ALL cell line. (a) Differentially expressed genes between J-gamma1 cells with or without knocking down FYB1 gene were shown by the volcanic plot. (b) Top 20 downregulated and upregulated genes after the knockdown of FYB1 gene in J-gamma1 cell line. (c) A total of 17 common genes between the RNA-seq data (1,051 differentially expressed genes (DEGs)) and Chip-seq data (213 SE associated genes in T-ALL) were identified. (d) ChIP-seq data from T-ALL cell lines showed that the promoter and enhancer region of IGLL1 had a consistent H3K27ac signal (track 1–7).

To reveal the potential targets of FYB1 responsible for T-ALL cell proliferation, we performed RNA-seq in J-gamma1 cells with or without knocking down FYB1 (GEO ID: GSE197450). About 1,051 DEGs were identified after knocking down FYB1 gene, including 757 downregulated genes and 294 upregulated genes (*P* < 0.05 and Log2 (fold change) >0.5 or Log 2(fold change) <−0.5, Figure 5(a) and *Supplementary 3*. IGLL1 was among the top 20 downregulated genes (Figure 5(b)) and was one of the 17 genes shared between DEG and SE-driven genes (Figure 5(c). Also, SE was observed crossing IGLL1 gene body in these 7T-ALL cell lines, determined by ChIP-Seq data (Figure 5(d), Track 1–7). Moreover, downregulated IGLL1 was confirmed after knocking down FYB1 by western blot (*Supplementary 2*). All these results point to IGLL1 as a potential downstream target of FYB1 signaling.

### 3.6. IGLL1 Knockdown Interferes with T-ALL Cell Proliferation and Promotes Apoptosis

**Figure 6:**
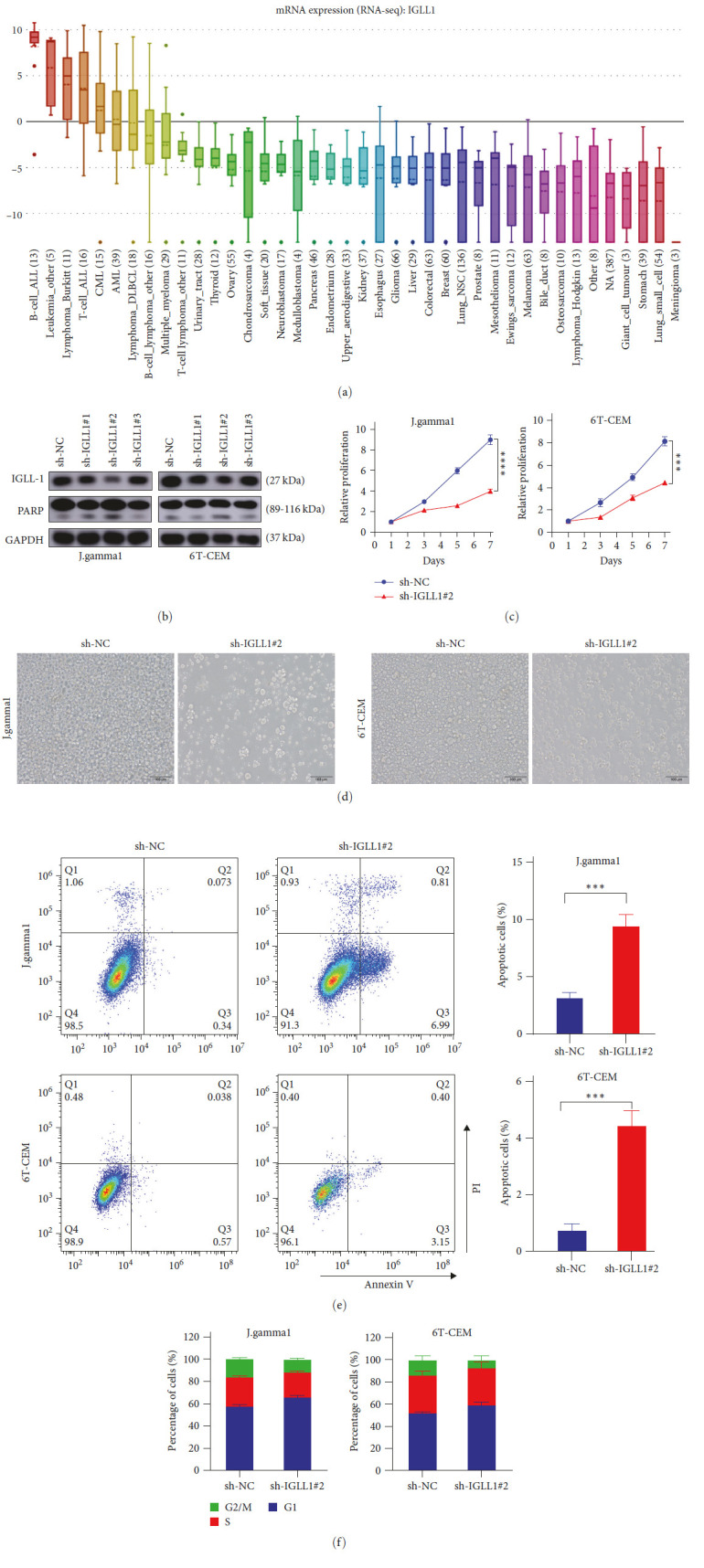
IGLL1 knockdown inhibited T-ALL cell proliferation and promoted apoptosis. (a) According to the CCLE (https://portals.broadinstitute.org/ccle), IGLL1 expression in T-ALL cells was higher than that in non-T-ALL cell lines. (b) The knockdown efficiency of IGLL1 in J-gamma1 and 6T-CEM cells was detected by the western blotting, and the expression of PARP was increased aftefr IGLL1 knockdown. (c) The knockdown of IGLL1 significantly inhibited the proliferation of J-gamma1 and 6T-CEM cell lines by cell proliferation assay. (d) The knockdown of IGLL1 significantly inhibited the proliferation of J-gamma1 and 6T-CEM cell lines by imaging assay. (e) The flow cytometry showed that the apoptosis rate of J-gamma1 and 6T-CEM cell lines increased after IGLL1 knockdown. (f) The knockdown of the IGLL1 gene inhibited the growth of J-gamma1 and 6T-CEM cell lines by affecting the cell cycle.

To further explore the role of IGLL1 in T-ALL, we analyzed the CCLE dataset (https://portals.broadinstitute.org/ccle) and found that the expression of IGLL1 was highly expressed in T-ALL (Figure 6(a)). To evaluate the biological characteristics of IGLL1, three independent shRNAs (Table 1) were used to knockdown IGLL1 in two T-ALL cell lines (J-gamma1 and 6T-CEM). As shown in Figure 6(b), efficient knocking down of IGLL1 was achieved by shRNA #2. We first assessed the effects of knocking down IGLL1, resulting in marked suppression of cell proliferation (Figure 6(c)). In agreement with this, decreased cell density was confirmed by imaging assay (Figure 6(d)), at least partially due to arrested cell cyle with more cells in G1 phase and less cells in G2/M phases (Figure 6(f)). Moreover, knocking down IGLL1 demonstrated synergistic effect with that of knocking down FYB1 (*Supplementary 2*). Besides, we also determined the effects of knocking down IGLL1 on the apoptosis and found enhanced apoptosis in J-gamma1 and 6T-CEM cells as a result of knocking down IGLL1 (Figure 6(e)). In addition, concomitant down regulation of PARP was also observed (Figure 6(b)). Taken together, these results showed that IGLL1 was an important regulator for the proliferation and survival of T-ALL cells.

## 4. Discussion

As an invasive hematological malignancy, T-ALL was characterized of heterogenous phenotypes and genetics. The past decades have seen improved treatment outcome, thanks to intensified combination therapy. However, around 20% of T-ALL patients would experience relapse and relapsed/refractory T-ALL patients have even worse prognosis [24–26]. Therefore, exploring the biology of T-ALL holds the promise to achieve a better therapeutic strategy for T-ALL.

Over the past decade, mounting evidence has shown that SE, associated with the pathogenesis of various solid tumors and hematological malignant tumors, plays a significant role in the regulation of key oncogenes [27–31]. In this study, we found that the expressions of several genes (CDK6, CCND3, ETV6, and FYB1) associated with T-ALL were driven by SE through exploring public H3K27ac ChIP-seq data. CDK6 serves as a key regulator of hematopoietic and leukemia stem cell activation [32], important for the survival of T-ALL cells. Genetically knocking out or pharmacological inhibition of CDK6 can prevent activated Notch signaling from inducing leukemia, indicating that CDK6 serves as a downstream effector of Notch signaling [33]. As being well-known drivers of cell cycle progression, D-type cyclins (D1, D2, and D3) are essential for tumorigenesis, which provides a basis for targeting therapy in tumors [34]. Reports have shown that CCND3 is very important for the proliferation and survival of ALL [35]. ETV6 (also known as TEL) gene, encoding a transcription inhibitor, plays a key role in hematopoiesis and embryonic development. Acting in a dominant negative manner, ETV6 mutations have been reported in various hematological malignancies in the reproductive system, including B-ALL as the most common one in children, and other hematological malignancies such as T-ALL, MDS, and AML [36, 37].

Acting as an essential adapter of the FYN and LCP2 signaling network, FYB1 is critical in bridging T-cell signaling to remodeling of the actin cytoskeleton and important for the fitness of normal T cell. However, whether FYB1 is essential for the fitness of T-ALL cells is largely unknown [12, 38].

Crucial for TCR signaling transduction, FYB1 has been reported for its involvement in the pathogenesis, invasion, and metastasis of various solid tumors [15–17]. However, there is no functional study in hematological malignant diseases. In this study, we found that T-ALL specific SE at FYB1 gene locus, highlighting that FYB1 might be transcriptionally driven by SE (Figure 2(a)). In line with this, FYB1 was highly expressed in T-ALL according to the CCLE database (Figure 2(b)). Moreover, we have confirmed that the expression of FYB1 protein in the T-ALL cell lines was much higher than that in the non-T-ALL cell lines (Figures 2(c) and 2(d)). These results suggested that high expression of FYB1 might be closely related to the proliferation of T-ALL cells and the occurrence and development of this disease. However, it is still unclear how FYB1 promotes the pathogenesis of T-ALL. Our study showed that downregulation of FYB1 effectively inhibited the growth of T-ALL cells both *in vitro* and *in vivo* by inhibiting cell proliferation and promoting apoptosis (Figures 3 and 4). In contrast, overexpression of FYB1 promoted the proliferation of T-ALL cells.

Among the top genes that were downregulated due to knocking down FYB1 and IGLL1 was also driven by T-ALL specific SE (Figure 5(d)). In consistence, IGLL1 was also highly expressed in T-ALL (Figure 6(a)). As a member of the immunoglobulin gene superfamily, upregulation of IGLL1 has been reported in many solid tumors [39, 40], but its function is largely unknown. In this study, we found that knocking down IGLL1 significantly inhibited the proliferation and growth of T-ALL cell lines by increasing the apoptosis rate and blocking its cell cycle. To sum up, all these results showed that IGLL1 was an important downstream effector of FYB1 and together with FYB1 was essential for the survival of T-ALL cells. Of course, more efforts, i.e., FYB1 ChIP-seq, are warranted to show the direct regulatory relationship between FYB1 and IGLL1.

Generally, our results indicate that FYB1-IGLL1 axis might play important roles in the pathogenesis of T-ALL, providing new insights for the biology of T-ALL. Given its role in controlling cell proliferation and apoptosis, FYB1-IGLL1 represents an interesting target for T-ALL therapy.

## Figures and Tables

**Table 1:** shRNAs used to knockdown FYB1 and IGLL1.

Name	Sequence
Homo-FYB1-sh1	CCGGCCAAATGTTGACCTGACGAAACTCGAGTTTCGTCAGGTCAACATTTGGTTTTTTGAATT

Homo-FYB1-sh2	CCGGGCTTCAAGCAAGGAGAGCAAACTCGAGTTTGCTCTCCTTGCTTGAAGCTTTTTTGAATT

Homo-FYB1-sh3	CCGGGCCATCTCTTCACAGTGTAAACTCGAGTTTACACTGTGAAGAGATGGCTTTTTTGAATT

Homo-IGLL1-sh1	CCGGGCCCAACAGCTGCATCGCAGACTCGAGTCTGCGATGCAGCTGTTGGGCTTTTTGAATT

Homo-IGLL1-sh2	CCGGTGAGGAGCTCCAAGCCAACAACTCGAGTTGTTGGCTTGGAGCTCCTCATTTTTGAATT

Homo-IGLL1-sh3	CCGGCGAAGGGAGCACCGTGGAGAACTCGAGTTCTCCACGGTGCTCCCTTCGTTTTTGAATT

## Data Availability

The data used and/or analyzed during the current study are available from the corresponding author on reasonable request (GSE197450).

## Supplementary Materials

Supplementary 1 Putative super enhancers identified in each of the 7T-ALL cell lines.

Supplementary 2 The downstream effectors of FYB1 gene.

Supplementary 3 RNA-seq results in J-gamma1 cell line with or without knocking down FYB1 (GEO ID: GSE197450).
