# Supplementary figures and images for: Super Enhancer Regulatory Gene FYB1 Promotes the Progression of T Cell Acute Lymphoblastic Leukemia by Activating IGLL1
Source: J Immunol Res. 2023 Sep 19;2023:3804605. doi: 10.1155/2023/3804605 (PMC10522422; doi:10.1155/2023/3804605)

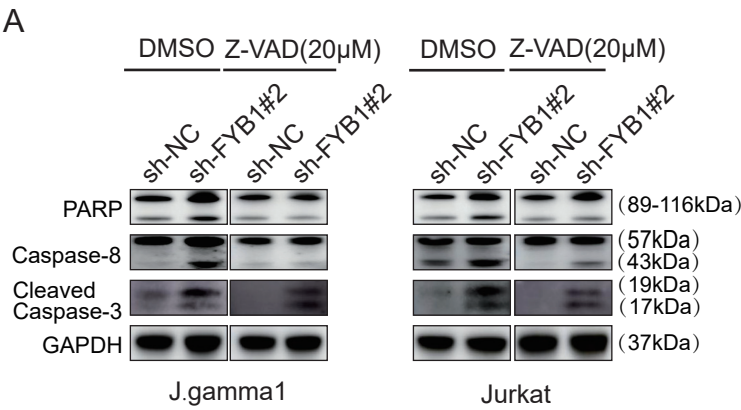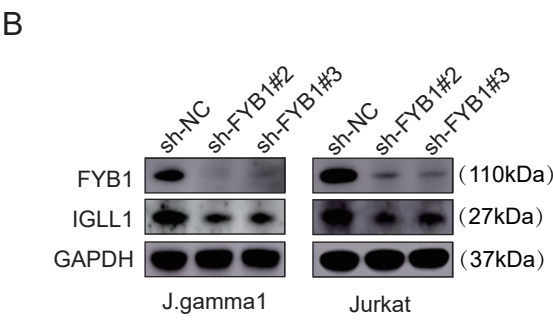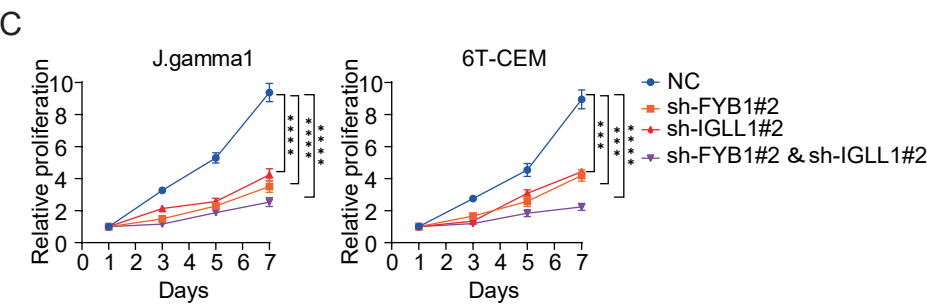

Supplement: Supplementary 2 — The downstream effectors of FYB1 gene. [file 3804605.f2.pdf]
